# Biomaterials in Cardiovascular Research: Applications and Clinical Implications

**DOI:** 10.1155/2014/459465

**Published:** 2014-05-08

**Authors:** Saravana Kumar Jaganathan, Eko Supriyanto, Selvakumar Murugesan, Arunpandian Balaji, Manjeesh Kumar Asokan

**Affiliations:** ^1^IJN-UTM Cardiovascular Engineering Centre, Faculty of Biosciences and Medical Engineering, Universiti Teknologi Malaysia, 81310 Johor Bahru, Malaysia; ^2^Rubber Technology Centre, Indian Institute of Technology, Kharagpur, West Bengal 721302, India; ^3^Department of Research and Development, PSNA College of Engineering and Technology, Kothandaraman Nagar, Dindigul, Tamil Nadu 624 622, India

## Abstract

Cardiovascular biomaterials (CB) dominate the category of biomaterials based on the demand and investments in this field. This review article classifies the CB into three major classes, namely, metals, polymers, and biological materials and collates the information about the CB. Blood compatibility is one of the major criteria which limit the use of biomaterials for cardiovascular application. Several key players are associated with blood compatibility and they are discussed in this paper. To enhance the compatibility of the CB, several surface modification strategies were in use currently. Some recent applications of surface modification technology on the materials for cardiovascular devices were also discussed for better understanding. Finally, the current trend of the CB, endothelization of the cardiac implants and utilization of induced human pluripotent stem cells (ihPSCs), is also presented in this review. The field of CB is growing constantly and many new investigators and researchers are developing interest in this domain. This review will serve as a one stop arrangement to quickly grasp the basic research in the field of CB.

## 1. Introduction

Last ten decades have shown tremendous growth in the field of material science and it is very synonymous to say that some materials have been used successfully to replace, assist, and repair some parts of the body and its functions. These materials widely called biomaterial. Numerous definitions for biomaterial by various scientists add some explored or unidentified scope to the definition. Some of the definitions of biomaterials are as follows. To begin with, Williams around 1987 stated “a nonviable material used in a medical device, intended to interact with biological systems” [1]. It was in 1999, Williams defined biocompatibility as “ability of a material to perform with an appropriate host response in a specific situation” [2]. This definition drastically changed the bird-view of biomaterials, until then that successful biomaterials played largely inert roles in the body. By the way, with respect to the above definition, that biomaterial not only provides some function, but also induces some biological responses. By accommodating the above and various considerations, a wide and distinct definition of biomaterials was made recently in Williams dictionary of biomaterials (2008) as follows: “ability of a biomaterial to perform its desired function with respect to a medical therapy, without eliciting any undesirable local or systemic effects in the recipient or beneficiary of that therapy, but generating the most appropriate beneficial cellular or tissue response to that specific situation, and optimizing the clinically relevant performance of that therapy” [3].

According to the recent reports of MarketsandMarkets, it has been forecasted that by 2017 the estimated global market for biomaterials will be 88.4 billion US$ with a compound annual growth rate (CAGR) of 15%. To add further, it has been estimated that Asian market is to grow at highest CAGR of 21.5% because of large number of people with disorders necessitating biomaterial-based medical products [4]. Nowadays, biomaterials are commonly used in various medical devices and systems: synthetic skin, drug delivery systems, tissue cultures, hybrid organs, synthetic blood vessels, artificial hearts, cardiac pacemakers, screws, plates, wires and pins for bone treatments, total artificial joint implants, skull reconstruction, and dental and maxillofacial applications [5–7]. Among various applications, the application of biomaterials in cardiovascular system is most significant. The use of cardiovascular biomaterials is projected to be predominant category of the biomaterials market in 2014, with a worth of about $20.7 billion [4].

The use of cardiovascular biomaterials (CB) is subjected to its blood compatibility and its integration with the surrounding environment where it is implanted [8]. Whenever, CB is used, two important considerations should be weighed equally; namely, (1) physical and mechanical features such as strength and deformation, fatigue and creep, friction and wear resistance, flow resistance and pressure drop, and other characteristics to be engineered must be considered and (2) biocompatibility or compatibility refers to material and tissue interactions are also to be considered. These characteristics have to be tested and appraised in an array of* in vivo* and* in vitro* experiments [9]. The first aspect mentioned is almost predetermined depending on the material chosen and its inherent properties. However, the second aspect biocompatibility is of immense interest for every cardiac implant material chosen and it decides the patency of the same. In the next subtitle, biocompatibility of cardiovascular biomaterials is discussed briefly before we move into the classification.

## 2. Biocompatibility of Cardiovascular Biomaterials

Cardiovascular biomaterials are used in two modes, namely, temporary and permanent [10]. Based upon mode of use, cardiovascular devices can be classified as temporary internal, temporary external, and permanent internal devices [11]. Testing of compatibility of cardiovascular devices largely depends upon the mode of use. All CB comes with contact with blood and this duration of contact further determines the testing parameters. Contact duration may be limited (less than 24 h), prolonged (>24 h to 30 days), and permanent (>30 days). Blood compatibility is to be evaluated for all blood contacting devices irrespective of the contact duration. According to ISO-10993, thrombogenicity, hemolysis, and immunology (complement activation) testing has to be performed for these devices [12, 13].

**Figure 1:**
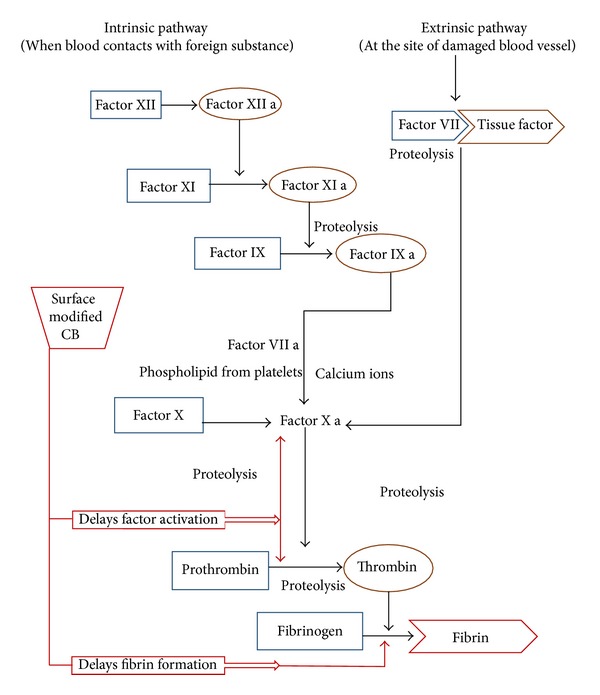
Blood clotting cascade: involvement of various clotting factors associated with intrinsic and extrinsic pathway.

One of the leading causes of cardiac biomaterial failure is the initiation of thrombosis formation by the blood contacting devices [14]. The mechanism underlying thrombus formation is discussed briefly for understanding the reason behind the cardiac device failure. Adsorption of proteins on the surface of implanted cardiac biomaterial through intrinsic pathway or through the release of tissue factor from the damaged cells at the site of injury (extrinsic pathway) is the initial event leading to thrombosis. The intrinsic pathway (Figure 1) is independent of injury and the adsorbed surface proteins form a complex comprising collagen, high molecular weight kininogen (HMWK), prekalikrein, and factor XII. Further, this complex got cleaved by contact activation and activates various immune responses like kinin system, coagulation, fibrinolytic, and complement system. Formation of clot due to intrinsic pathway system consists of series of conversion of inactive precursors to active form. To start with, activated factor XII activates factor XI which thereby activates factor IX and activated factor IX in turn activates factor X. Upon activation of factor X, it results in the cleavage of prothrombin to thrombin in presence of other supporting cofactors. Activated thrombin converts fibrinogen to fibrin which eventually gets stabilized as a red thrombus or clot. A damage to blood vessel endothelium releases tissue factor, collagen, and von Willebrand (vWF) which activates the extrinsic pathway. Tissue factors act as cofactor to activate factor X. There is also a possibility for factor VII to activate factor IX which in turn activate factor X. Except for factor VII, all factors of extrinsic pathway are similar to intrinsic pathway leading to the formation of thrombus [15, 16].

Hemolysis may result in impairment of oxygen carrying capacity of red blood cells (RBC). Hemolysis occurs when the RBC comes in contact with the material or its degradation products formed due to the shear stress generated because of relative motion between blood and the material surface. Moreover, to ascertain the various complement activation pathways immunology testing should be performed. ISO-10993 recommends direct contact* in vitro *C3a and SC5b-9 fragment activation using established testing methods such as an ELISA test [12, 13].

In addition to this, some toxic effects may also occur which is considered to be the components of biocompatibility and care should be taken to evaluate this depending upon the cardiac device. An ability to damage the system by means of chemicals is called toxicity. In higher organisms, toxicity may produce local and systemic effects. In local toxicity, the adverse effects emerge only in the affected areas, whereas in systematic toxicity the effects occur even far away from the distance of the application of the implant. Components of toxicity include cytotoxicity, genotoxicity, mutagenicity, and carcinogenicity. Cytotoxicity refers to the damage that occurs to the individual cells, that is, cell death due to necrosis or apoptosis. Genotoxicity refers to the alteration of the base pair sequence of the genomic DNA. The same genotoxicity when they are carried to the next generation through genes is known as mutagenicity. The alterations of the base pair sometimes lead to the generation of the malignant tumors referred as carcinogenicity [12, 13].

## 3. Classification of Cardiovascular Biomaterials

**Figure 2:**
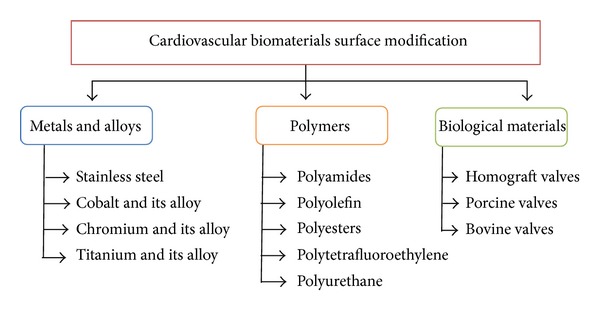
Classification of cardiovascular biomaterial.

Cardiovascular application of biomaterials (Figure 2) includes metals and their alloys, polymers, and some biological materials [17, 18]. In this subtitle, a brief discussion of these cardiovascular biomaterials for various applications will be highlighted.

### 3.1. Metals and Alloys

Metals have been used for more than a century in biomedical field [19]. The use of metals in cardiovascular applications includes heart valves, endovascular stents, and stent-graft combinations [20]. In majority of cardiovascular applications stainless steel, cobalt chromium (CoCr) alloys and titanium (Ti) alloys are widely used [19]. The basic properties of these common metallic CB are listed down under Table 1. One of the important applications of the CB is stents. Stents can be typically classified into three types based on their function and physical characters, namely, bare metal stents (BMS), drug-eluting stent (DES), and bioabsorbable stents. Metals and alloys utilized under these categories were discussed briefly to have a basic understanding of applications of metals as CB.

In the beginning, stainless steel used for implants contains vanadium, but it has been replaced with the advent of 18% Cr and 8% Ni alloy making it stronger for applications. Soon, addition of molybdenum (Mo) and reduction of carbon (C) made it corrosion resistant (316 L) and suitable for blood contacting devices like stent. Stainless steel is still the gold standard material for stent application in order to provide mechanical support to diseased arteries [21]. Further stainless steel is also widely used in heart valves, especially in making struts to support leaflets to avoid corrosion and provide mechanical strength to the valves.

Recently cobalt (Co) based alloys have gained entry for production of stents, although Co-based alloys were used in medicine since 1937. The use of Co-based alloys is highly preferred in coronary stent manufacturing because coronary interventionist demands for thinner struts which can be easily achieved by using the Co-alloys. since it has more strength compared with 316 L. Further, properties like nonferromagnetic and denser than stainless steel make driver cobalt alloy a feasible companion for making coronary stents. MP35N or cobalt-nickel-chromium-molybdenum (CoNiCrMo) alloys with a nickel content of 35% are used for cardiovascular pacing leads, stylets, and catheters. A new arrival cobalt-chromium-tungsten-nickel (CoCrWNi) alloy, also known as L-605, is used for making heart valves [22].

Titanium-based alloys (Ti-alloys) have wide acceptance and usage since 1970. The most commonly used Ti-alloys are commercially pure titanium (CP-Ti) and 5Ti-6Al-4V (titanium-aluminium-vanadium). One of the remarkable features of titanium is light weight. Density of Ti is only 4.5 g/cm^3^ compared to 7.9 g/cm^3^ for 316 stainless steel and 8.3 g/cm^3^ for cast cobalt-chromium-molybdenum (CoCrMo) alloys [23]. Moreover, Ti-alloys are known for their excellent tensile strength and pitting corrosion resistance suitable for cardiovascular applications. Another interesting feature of titanium alloys is shape memory effect possessed by the nickel-titanium (nitinol) alloys widely utilized for producing self-expanding memory stents [24, 25]. Although BMS have excellent mechanical characteristics, they failed because of serious limitations such as stent thrombosis which requires prolonged antiplatelet therapy and mismatch of the stent to the vessel size. Moreover the metallic stents impair the vessel geometry and obstruct side branches.

In order to rectify the complications present in BMS, drug-eluting stents (DES) was developed [26]. The DES were further classified into polymer free stents and metallic stents with polymer carrier to hold and release the drug. Drug-eluting stents basically consist of three parts: stent platform, coating, and drug. Some of the examples for polymer free DES are Amazon Pax (MINVASYS) using Amazonia CroCo (L605) cobalt chromium (Co-Cr) stent with Paclitaxel as an antiproliferative agent and abluminal coating have been utilized as the carrier of the drug. BioFreedom (Biosensors Inc.) using stainless steel as base with modified abluminal coating as carrier surface for the antiproliferative drug Biolimus A9. Optima (CID S.r.I.) using 316 L stainless steel stent as base for the drug Tacrolimus and utilizing integrated turbostratic carbofilm as the drug carrier. VESTA sync (MIV Therapeutics) using GenX stainless steel (316 L) as base utilizing microporous hydroxyapatite coating as carrier for the drug Sirolimus. YUKON choice (Translumina) used 316 L stainless steel as base for the drugs Sirolimus in combination with Probucol [27].

Another version of DES contains biosorbable polymers as a carrier matrix for drugs. Cypher, Taxus, and Endeavour are the three basic type of bioabsorbable DES. Cypher (J&J, Cordis) uses a 316 L stainless steel coated with polyethylene vinyl acetate (PEVA) and poly-butyl methacrylate (PBMA) for carrying the drug Sirolimus. Taxus (Boston Scientific) utilizes 316 L stainless steel stents coated with translute Styrene Isoprene Butadiene (SIBS) copolymer for carrying Paclitaxel which elutes over a period of about 90 days [28]. Endeavour (Medtronic) uses a cobalt chrome driver stent for carrying zotarolimus with phosphorylcholine as drug carrier [29]. Further some recent advances in the development of bioabsorbable DES resulted in the production of various new stent systems which includes BioMatrix employing S-Stent (316 L) stainless steel as base with polylactic acid surface for carrying the antiproliferative drug Biolimus. ELIXIR-DES program (Elixir Medical Corp) consisting both polyester and polylactide coated stents for carrying the drug novolimus with cobalt-chromium (Co-Cr) as base. JACTAX (Boston Scientific Corp.) utilized D-lactic polylactic acid (DLPLA) coated (316 L) stainless steel stents for carrying Paclitaxel. NEVO (Cordis Corporation, Johnson & Johnson) used cobalt chromium (Co-Cr) stent coated with polylactic-co-glycolic acid (PLGA) for carrying the drug Sirolimus [27]. Though DES is considered as a breakthrough in the development of stents; they are still associated with subacute and late thrombosis. Further, the polymer used as a vehicle for drug delivery may induce vessel irritation, endothelial dysfunction, vessel hypersensitivity, and chronic inflammation at the stent site.

Later they developed bioabsorbable stents to overcome the above-said problems of DES. Bioabsorbable stents stay for a limited period and promote healing of the blood vessel. The main purpose of the stent is to assist the arterial remodeling and this may take 6–12 months. Hence for a perfect cardiovascular application like stents, a wide varied biodegradable alloys have been experimented with a reasonable degradation life of 12–24 months [30–33]. This may overcome the need of unnecessary medication and also avoid late stent thrombosis. However, material for biodegradable stents is expected to meet some basic demands as it should be biocompatible and also its degradation products of the material must also be biocompatible. Finally, it should be able to stay in the place for several months before its complete bioabsorption and also its radial force of the resultant stent must be enough for scaffolding effect during the arterial remodeling period [30]. Based on these requirements, two metallic elements including iron and magnesium have been explored for this application [21]. Magnesium alloy stent is the first metallic bioabsorbable stent implanted in humans. Clinical evaluation conducted by Heublein et al. demonstrated higher degradation rates for Mg alloy from 60 to 90 days. Moreover, the stent was well integrated with both endothelial and smooth muscle cells indicating its overall biocompatibility [34].

### 3.2. Polymers

Polymers are the high molecular weight molecule made up of a small repeating unit called monomer. Polymers are widely utilized as implant material for cardiovascular application. Tailor made properties of polymers and better biocompatibility makes them an ideal choice when compared with metallic biomaterials [35]. The uses of polymers in cardiovascular application ranges from vascular grafts to stents, prosthetic heart valves, catheters, heart assist devices, hemodialyser, and so forth [35, 36]. In this section, various commonly used polymers for cardiovascular application are discussed (Table 2).

#### 3.2.1. Polyamides (PA)

PA is considered the first engineering thermoplastic invented in search of a “super polyester” fiber with molecular weights greater than 10,000. It is commonly called Nylon [37]. Application of polyamides includes transparent tubing's for cardiovascular applications, hemodialysis membranes, and also production of percutaneous transluminal coronary angioplasty (PTCA) catheters [35].

#### 3.2.2. Polyolefin

Polyolefins are the family of polymers consisting of a repeating unit of olefin or alkene in their polymeric chain. Polyethylene and polypropylene are the two important polymers of polyolefin have a wide range of medical application because of its better biocompatibility and chemical resistance [38]. In cardiovascular arena, both low-density polyethylene and high-density polyethylene are utilized in making tubing's and housings for blood supply. They are also utilized in production of blood bags to store blood. Polypropylene is used for making heart valve structures [17, 35].

#### 3.2.3. Polyesters

Polyesters are the family of polymers with ester functional groups. However, the term polyester was synonymously used for polyethylene-terephthalate (PET). Commercialization of PET was popular using the name Dacron. Synthesis can be done in many forms, but it is typically used as knitted or woven fabric for vascular grafts. Woven PET has smaller pores which reduces blood leakage and better efficiency as vascular grafts compared with the knitted one. PET grafts are also available with a protein coating (collagen or albumin) for reducing blood loss and better biocompatibility [39]. PET vascular grafts with endothelial cells have been searched as a means for improving patency rates [40]. Moreover, polyesters are widely preferred material for the manufacturing of bioabsorbable stents. Poly-L-lactic acids (PLLA), polyglycolic acid (PGA), and poly(D, L-lactide/glycolide) copolymer (PDLA) are some of the commonly used bioabsorbable polymers [27]. The Igaki-Tamai stent constructed from poly-L-lactic acid (PLLA) is the first absorbable stent implanted in humans. The clinical studies showed low complication rates for stent thrombosis [41]. The BVS everolimus eluting is another type of bioabsorbable stent coated with poly-D, L-lactide having metallic base has been using to carry the antiproliferative drug everolimus. The clinical records expressed lack of stent thrombosis and ensure total vascular function restoration [42, 43].

#### 3.2.4. Polytetrafluoroethylene

Polytetrafluoroethylene (PTFE) is synthetic fluorocarbon polymer with wide array of applications including biomedical industry. The most common commercial name of PTFE is Teflon by Dupont Co. Common applications of PTFE in cardiovascular engineering include vascular grafts and heart valves. PTFE sutures are used in the repair of mitral valve for myxomatous disease and also in surgery for prolapse of the anterior or posterior leaflets of mitral valves. PTFE is particularly used in implantable prosthetic heart valve rings. It has been successfully used as vascular grafts when the devices are implanted in high-flow, large-diameter arteries such as the aorta. Problem occurs when it is implanted below aortic bifurcations and another form of PTFE called elongated-PTFE (e-PTFE) was explored. Expanded PTFE is formed by compression of PTFE in the presence of career medium and finally extruding the mixture. Extrudate formed by this process is then heated to near its glass transition temperature and stretched to obtain microscopically porous PTFE known as e-PTFE. This form of PTFE was indicated for use in smaller arteries with lower flow rates promoting low thrombogenicity, lower rates of restenosis and hemostasis, less calcification, and biochemically inert properties [44–47].

#### 3.2.5. Polyurethanes

Polyurethane is a polymer formed by repeating units of urethane monomer. It is formed by the reaction between isocyanates with a polyol. Polyurethane backbone contains average two or more functional groups derived from the isocyanates and polyols. Polyurethane is widely used in cardiovascular application because of its good physiochemical and mechanical properties. Moreover, polyurethane is highly biocompatible which allows unrestricted usage in blood contacting devices. It has high shear strength, elasticity, and transparency. Moreover, the surface of polyurethane has good resistance for microbes and the thrombosis formation by PU is almost similar to the versatile cardiovascular biomaterial like PTFE. Conventionally, segmented polyurethanes (SPUs) have been used for various cardiovascular applications such as valve structures, pacemaker leads and ventricular assisting device [48]. Further SPUs can also be tailored to render biodegradable systems for the tissue engineering of vascular grafts and heart valves [49, 50].

### 3.3. Biological Materials

Metallic and polymeric materials serve as a better replacement in repair and replacement of cardiac tissues, but they fail in functional comparison with biological tissues. Sources of biological tissues range from human (allograft) to xenografts. Currently there are three types of bioprosthetic valves available commercially: allograft or homograft valves, porcine valves, and bovine pericardial valves [51]. Homografts are the intact human valves received from donors and cryopreserved as entire aortic or pulmonary roots. During implantation, it is further modified and adjusted to the size and shape of the recipient [52, 53]. Human donor is limited and xenograft tissues from bovine, porcine, and equine are explored. Pericardium isolated from porcine and bovine is used for making the bioprosthetic valve and it varies slightly depending upon the source. Collagen content is higher from bovine origin compared with porcine. Both porcine and bovine valve have displayed similar hemodynamics; however, bovine pericardial valves show less obstruction compared to porcine valve [54, 55]. Porcine xenograft valve composed of aortic valve from pig directly preserved in low-concentration glutaraldehyde solution. Porcine valves are suited for old patients and they do not require the anticoagulant regimen as necessitated by the mechanical valves. Calcification and degradation of valves are the common problems associated with bovine or porcine replacements. To overcome these issues, both types of the tissues are treated with some other chemical agents in order to minimize their tendency to calcify over the duration of implantation and thereby improve their longevity. The use of these natural biomaterials as bioprosthetic valves and vascular grafts has typically required some chemical or physical pretreatment aimed to preserve and also increase the resistance of the material against degradation, thereby promoting better compatibility, and reduced immunogenicity of the materials [56, 57]. The summary of few important properties of allograft and xenograft are listed in Table 3.

## 4. Surface Modification of Cardiovascular Biomaterials

**Figure 3:**
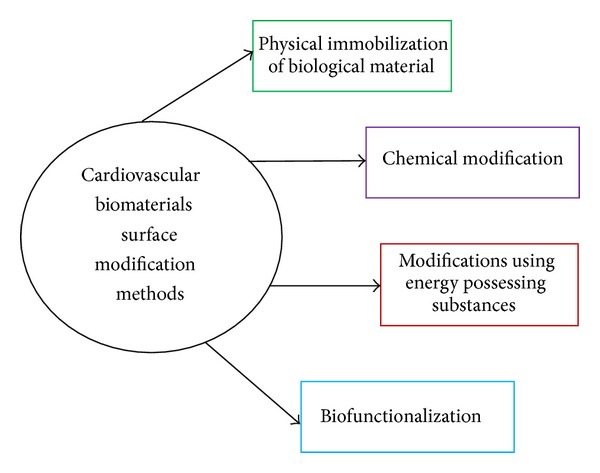
Cardiovascular biomaterials surface modification methods.

One of the major problems associated with cardiac biomaterial used for blood contacting devices is the compatibility of the materials with the blood. To circumvent the above, various strategies were adopted to modify the surface in order to improve the compatibility of the material. These modifications techniques may be broadly classified into three major classes; namely, physical immobilization of biological material, chemical modification, and modification of materials using energy possessing substances like plasma, ion implantation, and so forth were highlighted in the coming subsections (Figure 3).

### 4.1. Physical Immobilization of Biological Material

Physical immobilization of biological material is a technique utilizes any biological substance as a simple coating material without changing the structure of either. Activation and adhesion ability of the adhered biological material on the surface dictate the anticoagulation property of the implant. Most commonly used biological materials that are the proteins of human origin like heparin, fibronectin, collagen, vitronectin, and so forth have been found to improve the adhesion behavior of endothelial cells. Heparinization is one of the commonly used biological materials for modifying the surface of cardiovascular implants. Several methods have been used to immobilize heparin on different CB like 316 L SS, ePTFE, PET, and PU [58]. Concentration of heparin used on the implant surface has different effects on the blood and vascular cells, including endothelial cells (ECs) [59] and smooth muscle cells (SMCs) [60]. Even some direct thrombin inhibitors like hirudin and some of its derivatives like bivalirudin have shown to inhibit the active site of thrombin and thereby promote blood compatibility [61, 62]. Immobilization of cell-adhesive oligopeptide by covalent combination on the material surface has found to bind EC selectively [63]. Immobilization of other anticoagulant molecules like thrombomodulin (TM) and NO has also shown improved results. Coimmobilization of TM along-with urokinase and endothelial protein C receptor has been investigated by a group [64]. Utilization of NO as an immobilizing material resulted in the modulation of blood vessel tone, inhibition of platelet activation, and leucocyte adhesion and SMC hyperplasia [65]. Several trails involving the regulation of inflammatory system or complementary system by using some key players like apyrase [66], CD47 [67], and soluble complement receptor 1 [68] are under scrutiny.

### 4.2. Chemical Modifications

Chemical modifications introduce new molecules on the material surface through coupling, grafting, and coating. Diamond like carbon was used to coat the artificial heart valves (AHV) [69], ventricular assist devices (VAD), TiN coating for VAD, and Ti-O in metal stents [70]. SiC coating for cardiovascular implants resulted in decrease in platelet adhesion and also less inflammatory reactions. Diamond like carbon has similar advantages as SiC and also additionally it provides higher hardness and smoothness, lower frictional coefficient, chemical inertness, biostability, and also good blood compatibility making it an elegant choice for the applications on vascular stents, AHVs and VADs [71]. Another important chemical modification of cardiovascular implants is the use of polyethylene oxide (PEO) (–CH_2_–CH_2_–O)_*n*_ and the related molecule polyethylene glycol (PEG) with hydrophilic long chain (HO–[CH_2_–CH_2_–O]_*n*_–H). Anticoagulation properties of PEO are dependent on the length of the chain structure and it has been observed when the repeating unit is over 100 PEO displayed excellent blood compatibility [72]. Similarly PEG introduced surface presents excellent blood compatibility by providing adhesion resistance to small biomolecules like fibrinogen and cells such as platelets and leukocytes [73]. An interesting case to mention that is 2-methacryloyloxyethyl phosphorylcholine (MPC) polymer that was developed two decades ago is well known for its blood compatibility. It has been shown that this polymer surface could inhibit platelet adhesion and minimize the protein adsorption significantly resulting in wide-spread usage in cardiovascular devices like dialysis, heart pumps, or VADs [74].

### 4.3. Modifications Using Energy-Possessing Substances

This subsection delineates the various energy modification processes utilized for cardiovascular implants surface modification. Several modes of energy carrier modification are available. These modes alter the surface of the implant and also facilitate the addition of coatings to improve the blood compatibility. Plasma surface modification is one of the widely utilized techniques to improve blood compatibility of cardiovascular implants. Air plasma modified segmented polyurethane holds good for vascular graft application [75]. PET films grafted with acrylic acid was modified using oxygen plasma before immobilizing with heparin was found to improve the blood compatibility [76] and also with collagen to facilitate the growth of smooth muscle cells [77]. Another important mode of modification is using UV-exposure. PTFE and PET exposed to UV-irradiation have been found to facilitate the endothelial and smooth muscle cell adhesion favorably compared with the unexposed polymers [78]. Similarly laser treatments have also been found to be exquisite tool to improve the blood compatibility by providing antithrombogenic surface [79]. Microwave treatment has also conjoined this race and it has been efficient in producing blood compatible surface on some polymers used for cardiovascular implants [80]. Ion implantation utilizes precisely controlled ion species and doses to modify the surface. Recent investigations implied that ion implantation can improve the wettability and anticoagulant nature of polypropylene and polystyrene and thereby promoting endothelial cell adhesion [81, 82].

## 5. Biofunctionalization of Cardiovascular Biomaterials

Endothelium is the layer which surrounds the entire vasculature ranging from heart to minute capillaries. A vast amount of research is instituted to make endothelial surface on the cardiovascular implants to mimic the natural environment for better biocompatibility. Endothelial cells are end-differentiated cells which are not capable of cell dividing and expansion [83]. Several strategies were employed to promote anticoagulant or endothelial promoted surface modification by impregnating the surface with active molecules or vascular cells seeding but due to cost and time involved in this process outweighed this technology. With the advent of endothelial progenitor cells (EPC) in 1997, rapid self-endothelization developed extensively paving the way for various novel methods for* in vivo* endothelization on the surface of cardiovascular implants especially vascular grafts and stents [84]. EPCs are the relatively small population of CD34^+^ circulating mononuclear cells in the circulatory system available as two forms, namely, early EPC and late EPC. Based on these two forms, EPC have been exploited in two different ways: one way is to construct and immobilize the early EPCs at the site of injury which will secrete angiogenic cytokines which will flourish the resident ECs and the late EPCs. Another way is to construct the surface with late EPCs which in turn promotes neoangiogenesis and repair the damaged site by their native ability to proliferate at high rate [85, 86]. As previously stated several active molecules such as vascular endothelial growth factor (VEGF), stromal derived factor-1 (SDF-1), nerve growth factor (NGF), and granulocyte-colony stimulating factor (G-CSF) were utilized to induce neovascularization and repair the injury [87]. A recent study utilizing NGF-bound vascular grafts showed significant immobilization of EPC and a similar preparation using SDF-1/heparin found to recruit both EPCs and smooth muscle progenitor cells tackling the two important issues, namely, endothelization and remodeling of blood vessels [88, 89]. EPC capture technology is the way through which circulating EPC is captured by using anti-CD34^+^that was impregnated on the surface of stents. Genous R-Stent is the first medical device utilizing this technology [90] and various clinical investigations evaluated this device comprehensively. One of the studies postulated that this EPC capture technology was feasible and safe for primary percutaneous coronary intervention for STEMI without the incidence of late stent restenosis [91]. In another independent trial, coronary stenting with the Genous resulted in good clinical outcomes and low incidences of repeat revascularization and stent thrombosis [92]. However, some recent evaluation came in contrast to the above findings, where they reported higher risk of restenosis while using Genous compared to drug-eluting stents [93]. To add further, Genous stent used in a population of elderly patients resulted in a significantly higher target vessel failure rates compared with younger patients. Moreover, target lesion revascularization rates were higher with increasing age and there was no difference in stent thrombosis [94]. Another worthy research to mention is the use of exponential enrichment technology in which DNA-aptamers with a high affinity to EPCs were identified. These EPC specific molecules are grafted on the surface of polymer disk [95], stents [96], and Ti-implants [97] found to attach EPCs to the aptamer-coated implants. This selective adhesion of EPCs promoted endothelial wound healing and also decreased the neointimal hyperplasia to a certain extent.

Recent researches utilize various cell sources for treatment of cardiovascular diseases. Human embryonic stem cells (h-ESC), mesenchymal stem cells, endothelial progenitor cells, and induced human pluripotent stem cells (ihPSCs) are some of the cell sources explored for treatment of cardiovascular diseases. The most versatile ihPSCs are induced from human somatic cells like fibroblast by transfecting with stem cell associated genes which then exhibit the characteristics of h-ESCs. Human-iPSCs are preferred for cardiomyocytes differentiation used for autologous cardiomyocyte transplantation therapy since they provide a better alternative to human embryonic stem cells (hESCs) derived cardiomyocytes (hESC-CMs) for two reasons. The first reason is that ihPSC-CMs overcome the political and ethical problems of damaging the human embryos; and secondly it helps to achieve a considerable quantity of patient specific cardiomyocytes that could be derived from ihPSCs, thus allowing the use of autologous cardiomyocytes for transplantation [98]. Recently concluded researches explored cardiomyocytes differentiated from ihPSC for long QT syndrome. They isolated patient-specific cells through skin biopsies, reprogramming their cells into iPSCs and then differentiating those iPSCs into cardiac cells. They were successful in treating both long QT syndrome of types 1 and 2 using these differentiated cardiomyocytes [99, 100]. Further iPSCs act as a source material to generate cardiac patch or tissue through cell sheet technique. Tulloch et al. produced engineered heart tissues (EHT) from hESC and ihPSC by using a collagen I-based method and a commercially available system from FlexCell [101]. Moreover, several groups had shown that iPSCs can be differentiated in to endothelial and vascular smooth muscle cells which highlight the potential of these cells for vascular generation and understanding the mechanism of diseases such as hypertension, coronary heart disease, and diabetic cardiomyopathy [102–106].

## 6. Conclusion

Cardiovascular biomaterials were projected to be predominant category of the biomaterials market in 2014, with a worth of about $20.7 billion. Major problem associated with CB is the blood compatibility and various standards have evolved in evaluation of biocompatibility of CB. This ensures the quality of biomaterials used in the cardiovascular applications. CB falls mainly into three categories, namely, metals, polymers, and biological materials. Properties of these materials limit their use in various applications. Polymers have emerged as a versatile choice for various cardiovascular applications. However, the problem of blood compatibility is still a major issue and several surface modifications are adopted to circumvent this and to develop biocompatible CB.

The field of cardiovascular biomaterials have shown a growth in the past two decades, but it still lacks more basic experiments and clinical data that have to be generated extensively by researchers to augment this area. To list, still there is a need to develop materials that mimic the properties of the natural cardiac tissues and this can be done by producing composite materials that exhibit the property of both natural and synthetic materials. Further some novel surface modification strategies should be evolved to develop better biocompatible cardiac biomaterials. This can be achieved by doing research in the field of endothelization and also utilizing nanotechnology as a tool to modify the CB surfaces.

## Figures and Tables

**Table 1:** Relative differences between the three commonly used metallic alloys as cardiovascular biomaterials.

Properties	Stainless steel	Co-Cr alloys	Ti-alloys
Stiffness	Best	Better	Good
Strength	Better	Good	Best
Corrosion resistance	Good	Better	Best
Blood compatibility	Good	Better	Best

**Table 2:** Properties of polymers utilized as cardiovascular biomaterials.

Properties	Polyamides	Polyolefin	Polyester	Polytetrafluoroethylene	Polyurethanes
Strength	Medium	Good	Good	High	Better
Hardness	Medium	High	High	High	Medium
Rigidity	Medium	High	High	High	Medium
Blood compatibility	Good	Better	Moderate	Low	Good

**Table 3:** Characteristics of biological materials used as cardiovascular biomaterials.

Properties	Allograft	Xenograft
Strength and durability	Moderate	Moderate
Blood compatibility	Best	Better
Blood flow dynamics	Better	Better
Corrosion resistance	Moderate	Moderate
